# Effect of combined psycho-physiological stretching and breathing therapy on sexual satisfaction

**DOI:** 10.1186/1471-2490-13-16

**Published:** 2013-03-25

**Authors:** Roohallah Bay, Shaiful Bahari Ismail, Wan Mohd Zahiruddin, Wan Nor Arifin

**Affiliations:** 1 Department of Family Medicine, Universiti Sains Malaysia, Kentalan, Malaysia; 2 Department of Community Medicine, Universiti Sains Malaysia, Kentalan, Malaysia; 3 Unit of Biostatistics and Research Methodology, Universiti Sains Malaysia, Kentalan, Malaysia

**Keywords:** Psycho-physiological therapy, Stretching therapy, Breathing exercise, Sexual satisfaction

## Abstract

**Background:**

During the last few decades, marital tensions and stresses have influenced various dimensions of life. The objective of the current study was to examine the effects of combined psycho-physiological therapy (stretching therapy combined with breathing exercise) on sexual satisfaction among heterosexual men.

**Methods:**

For this research, we used “convenience sampling” to select 80 males, who were then split equally into two groups, the intervention group and the control group, both groups containing men who had voiced a desire to be in the experimental group. For collection of data, we used an identical quasi-experimental design called the “nonequivalent control group.” Therapy sessions, each lasting 90 to 120 min, were carried out on the same 3 days of the week (Sunday, Tuesday, and Thursday) for a total of 20 sessions. The volunteers were selected from heterosexual men with stable relationships, who had been married a minimum of 6 months and were ages 20 to 55 years of age. Pre-tests, post-tests, and follow-up tests were conducted in a clinic at the Hospital Universiti Sains Malaysia (HUSM ^[1]^ ). For assessment, we used the sexual satisfaction subscale of the ENRICH ^[2]^ questionnaire.

**Results:**

The intervention group had better post-test scores than the control group. Also, follow-up test scores for the intervention group were marginally better than those for the control group, but the difference did not reach statistical significance.

**Conclusions:**

Combined psycho-physiological therapy including stretching and breathing exercise leads to improved sexual satisfaction.

## Background

During the last few decades, marital tensions and stresses have influenced various dimensions of life. Studies done on married persons have shown that they eat healthful food, have healthy bodies and suffer less from psychological difficulties [1]. One major cause of day-to-day arguments among couples is often the need for intimate contact. That is not to say that sexual intercourse is a panacea for all problems couples encounter in life. However, the satisfaction obtained from intimate contact, inclusive of some forms of physical contact, is fundamental to personal health and, in fact, to human nature. Sex is an individual psychological event, and the attitudes of the individuals can have a major impact on sexual activity, with earlier experience, anxiety, fatigue and overwork all potentially having a major adverse impact [2]. Thus, training is necessary to teach couples to react appropriately to life’s events and to give attention to family, marital, and sexual relationships.

From the early 1980’s, psychotherapy has grown rapidly and now offers the best approach to a better & more complete treatment model [3]. Psycho-physiological sex therapy is a new alternative and complementary therapy designed to save marriages and increase societal welfare. Sex therapy is the result of relatively recent scientific attention to the human sexual function and its dysfunctions. Increased knowledge of the physiology and the psychology of human sexual behavior has led to a new professional appreciation of the human sexual response. At a time when sexuality is being more openly discussed in society, we are beginning to realize how uninformed many people really are about this important personal topic. Depending on the issue being addressed, sex therapy can be helpful for individuals or couples. Some issues can be dealt with on an individual basis while others are best treated in the context of the relationships between couples [4].

The current research investigated the effectiveness of combined psycho-physiological therapy (stretching therapy and breathing exercise) on the sexual satisfaction in heterosexual men. Currently, stretching therapy is increasing in the United States, where 10–15 million people participate in stretching classes [5]. In 1997, more than 40% of the people living in the United States practiced at least one type of complementary or alternative therapy, psycho-physiological stretching therapy being one of those modalities [6]. In addition, 65% of people receiving mental health services engaged in one type of alternative or adjunctive therapy [7]. The research findings have described the possible beneficial effects of stretching on mental health. Specifically, the literature suggests that some style of therapy, such as yoga, may be related to increases in coping skills and self-esteem and to decreases in stress, anxiety, and depression [8-11].

Research has also shown that some kind of breathing exercise, such as Bhasrika, helps in treatment. These breathing exercises use the abdominal muscles and the diaphragm to put pressure on the internal organs. In addition, breathing exercises affect the mind, the brain and the nervous system, they increase oxygen levels in the blood, and they reduce carbon-dioxide levels in the blood. Thus, regular breathing exercises ensure proper oxygenation of all parts of the body, thereby helping to purify the blood, to remove toxins and carbon dioxide from the body, and to possibly cure many diseases [12].

Stretching and improved flexibility have been important goals in both the recreational and the therapeutic fields in the belief that it is beneficial to promote good physical and mental function [13-16]. Stretching is thought to reduce the risk of injury, relax hypertonic muscles, lengthen shortened tissue [17,18], and improve faulty posture [14,19,20]. In spite of its constant use and clinically observable results, the research on how effective stretching is and how stretching achieves its effects remains somewhat controversial. Utilizing various movement philosophies, for example, proprioceptive neuromuscular facilitation (PNF) [21], the Feldenkrais method [22], Pilate’s method [23], and Tai Chi [24], can help to restore or improve flexibility, decrease pain, and improve coordination, thereby improving overall function. This article and ensuing articles by different authors will attempt to investigate and explain some basic facts, philosophies and observations regarding flexibility, the reasons for flexibility being of such clinical importance, and the manner in which flexibility affects well-being.

This study had one main goal and two secondary goals. The main goal of this research was to evaluate the effect of combined psycho-physiological therapy (stretching therapy and breathing exercise) on the sexual satisfaction among heterosexual men and to determine if differences in sexual satisfaction exist between heterosexual men who undergo combined psycho-physiological therapy and those who do not. The two secondary objectives were to determine if any of the observed differences between the sexual satisfactions of heterosexual men who underwent combined psycho-physiological therapy and those who did not were influenced, either individually or as a whole, by (1) age or (2) education level.

## Methods

### Inclusion/exclusion criteria

In this research, the independent variable was combined psycho-physiological therapy (stretching therapy and breathing exercise), and the dependent variable was the sexual satisfaction of the heterosexual men. The therapy schedule, the gender (only male), co-morbidities (stable, non-cardiac and no prostatic disease), social, economic and cultural factors, the location and the environment of the therapy, and the therapist were controlled variables. Moderator variables were the education level and the age. For the analysis of the effect of age, the subjects were placed into two age groups: 20–35 and 36–55 years of age.

### Intervention therapy methods

For this research, we used static stretching and breathing exercise techniques to form an integrated therapy. Static stretching involves a muscle that is slowly and passively stretched to full range, and continued tension is then maintained for an extended period of time, e.g., 15 ± 2 min, to further increase the muscle’s length. For maximum gain, it is important that the person stretching remain in the assumed position until the muscle relaxes.

Each treatment session started with a 10-minute warm up and rhythmic breathing (10-sec inhaling, 25-sec holding of the breath, 15-sec exhaling, and 10-sec holding of the breath), after which stretching therapy started. Each muscle was stretched separately, and all the surfaces of the muscles were stretched. Patients exhaled when a muscle was stretched, and patients inhaled when a muscle was relaxed. Each session finished with rhythmic breathing techniques and a 5-minute cooling down. In this research program, we consider 20 sessions of 90 to 120 minutes each.

### Materials and research methodology

Eighty (80) heterosexual men were selected from volunteers who had registered at the Hospital Universiti Sains Malaysia’s (HUSM') clinic in Kota Bharu (Kelantan, Malaysia). These 80 men were divided into two equal groups, the intervention group and the control group, both groups consisting of some men who had voiced a strong desire to be in the intervention group and some men who had voiced no preference. Patients with cardiac diseases, uncontrolled type-1 and −2 diabetes, HbA1c < 8.5%, prostatic diseases, major uncontrolled psychiatric disorders, chronic arthritis, histories of alcohol or drug abuse and clinically significant baseline laboratory abnormalities were excluded. To be included, the volunteers had to be heterosexual men with stable relationships, who had been married for a minimum of 6 months and who were 20 to 55 years of age; signed informed consent was also required.

We used “convenience sampling” for the selection. The volunteers were selected from among men who visited the HUSM’s Family Clinic. For data collection, we used the quasi-experimental design called the “non-equivalent control group.” Pre-tests were done on the individuals of both groups before the intervention began, post-tests were done on the individuals of both groups after the intervention had been completed, and follow-up tests were done on the individuals of both groups one month after the last session.

For the setting, we used a hall nearby HUSM’s sport complex. Each week, the intervention group underwent the combined therapy on the same three days (Sunday, Tuesday, and Thursday) for 90 to 120 minutes each day for a total of 20 sessions. The member of the control group were left to follow the usual routines of their lives.

### Procedure

We announced this research at the hospital and the clinics of Universiti Sains Malaysia (Kelantan). Then, to obtain information about the volunteers’ ages, genders, drugs used, education levels, other illnesses that they suffered, etc., we asked the volunteers for this research to complete a questionnaire. This information was necessary both to form similar groups to prevent intervener variable effects bias and to manage the controlled and the moderator variables, as well as the obstructive factors.

Initially, 150 heterosexual men volunteered to participate in this research. Then, by paying attention to the control and the moderator variables and trying to decrease interviewer bias, we invited the 150 men for interviews and checkups. At the time of the interviews, a researcher obtained information on the patients’ backgrounds, durations of disease, types of medicines used, nutrition with lists of daily meals, etc. Then, according to the research design (unequal control groups), we made one intervention group and one control group. Ultimately, from the 100 patients selected, we divided 80 men into two equal groups of 40 men each.

For the intervention group’s patients, we conducted combined psycho-physiological therapy (stretching therapy and breathing exercise). The duration of each session for the intervention group was around 90 to 120 minutes, and we conducted three sessions per week on the same three days (Sunday, Tuesday, and Thursday) for a total of 20 sessions. The control group’s patients were allowed to continue their daily routines. It is important to note that all of the volunteers (both those in the control and the intervention groups) had gone to the HUSM clinic for medical checkups and that before the first therapy session, all of the volunteers had undergone pre-tests to establish a baseline. None of them claimed to have had or to have been treated for any sexual dysfunction. After the last therapy session, all of the volunteers also underwent post-tests to establish any changes in the levels of sexual satisfaction. After the last therapy session, the members of the intervention group were also allowed to return to the usual routines of their daily lives. About one month later, on a specified day, we performed follow-up tests on all patients of both groups to determine the long-term effects of the therapy.

### Measured parameters

In order to assess sexual satisfaction, we used a subscale of the ENRICH questionnaire. The Couple Sexual Relationship Scale is taken from the PREPARE/ENRICH Inventory and contains ten items. The total score is the sum of positive and negative items. The range of scores is from 10–50. The mean and the scoring levels were based on a national sample of 50,000 married couples with data on ENRICH described in a book by David Olson, Amy Olson-Sigg, and Peter Larson (*The Couple Checkup: Find your relationship strengths*. Nashville, TN: Thomas Nelson (2008)). The ENRICH scales were developed through extensive theoretical & empirical analyses [25].

### Ethical approval

This study was conducted with the approval of the Human Research and Ethics Committee, USM.

### Statistical methods

Data entry and statistical analyses were done using SPSS version 19. In the descriptive statistics, we used the mean and the standard deviation (SD), as well as tables and charts. In inferential statistics, in order to compare the data, we used Repeated Measure ANOVA. For assumption checks, we examined the residuals for the normality and the equality of variances. On examination of the histograms of residuals, all were found to be normally distributed. The spreads of the error variances at each level looked similar, so the assumption of equal variances was met. Further, the Levene’s test was not significant, so equal variances were assumed.

## Results

All data showed a P-values on Mauchly's Test of more than 0.050 (0.086), so we had to assume sphericity when addressing within-subject effects. As Table 1 shows, the P-value for time is 0.858, which means that some changes had occurred with passing time, but those changes were not statistically significant. In Table 1, the time and group P-value is significant (0.004), which means that some statistically significant changes had occurred within the groups with passing time. Also, as Table 2 shows, the group P-value of 0.140 means that no statistically different changes had occurred between the groups regardless of time.

**Table 1:** Tests of within-subject effects

**Source**		**df**	**F**	**Sig.**
Time	Sphericity Assumed	2	0.154	0.858
Time * Group	Sphericity Assumed	2	5.652	0.004
Time * Group * Age	Sphericity Assumed	2	0.975	0.380
Time * Group * Education	Sphericity Assumed	2	2.540	0.083

**Table 2:** Tests of between-subjects effects

**Source**	**df**	**F**	**Sig.**
Group	1	2.228	0.140
Group * Age	1	0.001	0.972
Group * Education	1	0.091	0.763

In order to establish the amount of change, we analyzed the details of the estimated marginal mean data. As Table 3 shows, the estimated marginal mean data for the intervention group show a pre-test sexual satisfaction value of 34.108, a post-test value of 37.852, and a follow-up test value of 36.212. Simultaneously, the control group’s estimated marginal mean data show a pre-test sexual satisfaction value of 39.286, a post-test value of 36.598, and a follow-up test value of 37.813. Comparing the post-test mean score of the intervention group (37.852) with that group’s pre-test lower to upper bound range (31.978- 36.238), the post-test change for the intervention group is found to be significant, but the same is not true for the control group, which means that the sexual satisfaction of the intervention group was improved by the combined psycho-physiological therapy. In the same table (Table 3), a comparison of the follow-up test mean score of the intervention group (36.212) with that group’s pre-test and post-test lower to upper bound ranges (31.978-36.238 and 36.011-39.692) shows that the changes were not statistically significant, which means the intervention group improved while following the combined psycho-physiological therapy, but one month after completing the therapy, the improvement had disappeared.

**Table 3:** Effects of group and time

**Group**	**N**	**Time**	**Mean**	**Std. error**	**95% Confidence interval**	
					**Lower bound**	**Upper bound**
Intervention	40	1	34.108	1.068	31.978	36.238
		2	37.852	0.922	36.011	39.692
		3	36.212	0.976	34.265	38.159
Control	40	1	39.286	1.433	36.427	42.144
		2	36.598	1.238	34.129	39.068
		3	37.813	1.309	35.200	40.425

Time 1 = pre-test, time 2 = post-test, and time 3 = follow-up test.

As Table 1 shows, the time, group and age P-value is 0.380, which means that age had some effect on the groups with passing time, but the effect was not significant. Also, Table 2 shows that the group and age P-value (between groups) was not significant (0.972), which means that regardless of time, statistically age had no effect on the groups.

In order to quantify the effects of group, age, and time, we analyzed the details of the estimated marginal mean data. As Table 4 shows, the intervention group’s estimated marginal mean data for age group 1 (20–35 years of age) show a pre-test sexual satisfaction value of 33.071, a post-test value of 36.611, and a follow-up test value of 36.175. For age group 2 (36–55 years of age), the values are 35.145, 39.092, and 36.250, respectively. Simultaneously, the control group’s estimated marginal mean data for age group 1 (20–35 years of age) show a pre-test sexual satisfaction value of 39.750, a post-test value of 34.500, and a follow-up test value of 37.000. For age group 2 (36–55 years of age), the corresponding values are 38.821, 38.696, and 38.625.

**Table 4:** Effects of group, age, and time

**Group**	**Age (years)**	**N**	**Time**	**Mean**	**Std. error**	**95% Confidence interval**	
						**Lower bound**	**Upper bound**
Intervention	20-35	19	1	33.071	1.198	30.681	35.462
			2	36.611	1.035	34.546	38.676
			3	36.175	1.095	33.990	38.360
	36-55	21	1	35.145	1.767	31.618	38.672
			2	39.092	1.527	36.045	42.139
			3	36.250	1.615	33.027	39.473
Control	20-35	13	1	39.750	2.568	34.626	44.874
			2	34.500	2.218	30.073	38.927
			3	37.000	2.347	32.317	41.683
	36-55	27	1	38.821	1.271	36.286	41.357
			2	38.696	1.098	36.506	40.887
			3	38.625	1.161	36.308	40.942

Time 1 = pre-test, time 2 = post-test, and time 3 = follow-up test.

In Table 1, the time, group and education P-value is 0.083, which means the education level had some effect on the groups with passing time, but the effect was not statistically significant. Also, in Table 2, the group and education P-value (between groups) is not significant (0.763), which means that regardless of time, statistically, education had no effect on the groups.

In order to quantify the effects of group, education, and time, we analyzed the details of the estimated marginal mean data. As Table 5 shows, the intervention group’s estimated marginal mean data show that education group 1 (primary-secondary education) had a pre-test sexual satisfaction value of 34.895, a post-test value of 36.953, and a follow-up test value of 36.389. For education group 2 (college/university-level education), the corresponding values are 33.321, 38.750, and 36.036. Simultaneously, the control group’s estimated marginal mean data for education group 1 (primary-secondary education) show a pre-test sexual satisfaction value of 38.446, a post-test value of 37.821, and a follow-up test value of 36.375. For education group 2 (college/university-level education), the corresponding values are 40.125, 35.375, and 39.250.

**Table 5:** Effects of group, education, and time

**Group**	**Education**	**N**	**Time**	**Mean**	**Std. error**	**95% Confidence interval**	
						**Lower bound**	**Upper bound**
Intervention	Primary-secondary	25	1	34.895	0.962	32.975	36.814
			2	36.953	0.831	35.295	38.612
			3	36.389	0.879	34.634	38.143
	College/university-level	15	1	33.321	1.906	29.518	37.125
			2	38.750	1.647	35.464	42.036
			3	36.036	1.742	32.560	39.512
Control	Primary-secondary	27	1	38.446	1.070	36.312	40.581
			2	37.821	0.924	35.978	39.665
			3	36.375	0.977	34.425	38.325
	College/university-level	13	1	40.125	2.658	34.821	45.429
			2	35.375	2.296	30.793	39.957
			3	39.250	2.429	34.403	44.097

Time 1 = pre-test, time 2 = post-test, and time 3 = follow-up test.

## Discussion

Approximately 35% of all family medicine departments offer some kind of instruction in alternative therapies. According to Bricklin, approximately 40% of the population of the United States (U.S.) is found to be using alternative therapies [26]. As popular interest in alternative medicine has increased, so has advanced practical nurse involvement, research attention, and the likelihood of insurance reimbursement. Research interest in alternative therapies at the National Institute of Health of the U.S. is growing. The Organization of Alternative Medicine (OAM) has recently funded a study of alternative therapies at several academic centers, including, among others, Harvard, Stanford and Columbia medical schools. Meanwhile, managed care organizations and insurance companies, including Mutual of Omaha, Blue Cross/Blue Shield of Washington and Alaska, and U.S. Health Care, are offering special health plans that include alternative therapies [26].

We hypothesized that combined psycho-physiological therapy (stretching therapy and breathing exercise) would have an effect on self-reported sexual satisfaction, and we designed this research to test that hypothesis. We found that the combined psycho-physiological therapy group achieved greater improvements in sexual satisfaction than the control group, but this difference didn’t remain significant on follow-up tests, which means that when following the therapy, patients experienced improved sexual satisfaction, but that improvement did not necessarily continue once therapy had been discontinued. Overall, these findings provide support for the beneficial effects of combined psycho-physiological therapy (stretching therapy and breathing exercise) on sexual satisfaction for 20- to 55-year-old heterosexual men. The data also show that neither age nor education had any significant effect on the benefits of psycho-physiological intervention.

Arpita suggested that stretching and breathing exercise such as yoga might lead to a physiological balance, a decrease in psychological distress, and an increase in self-esteem [8]. Vahia, Vinekar, and Donngaji [27] studied 30 hospital patients with a range of diagnoses including depression, peptic ulcers, schizophrenia, and anxiety reaction disorder. Treatment consisted of stretching postures, philosophy classes, breathing techniques, and meditation for an average of four to six weeks. Patients attended classes six days a week for one-half hour to an hour each day. Results indicated that patients suffering from anxiety and depressive disorders showed a decrease in symptoms.

Our study also showed different results for the intervention and the control groups. The combined therapy had a significant effect, and this result was independent of education level and age. Thus, our study supports a link between alternative healing therapies and an increase in mean sexual satisfaction. This creates a need for structured programs teaching lifestyle changes, non-pharmacologic interventions and alternative therapies in conjunction with conventional treatments. Alternative therapies can be extremely useful adjuncts to conventional care, and they sometimes provide the most appropriate treatment for conditions such as the lack of sexual satisfaction, erection disorders, and the lack of sexual desire.

The patient’s psychology has always been important. If lifestyle changes and alternative healing, such as stretching therapy combined with breathing exercise, are taught to patients, the risk of sexual problems and the effects of sexual problems on family life will decrease. Pharmacologic treatment is an area of expertise that requires special attention in patients with family problems. However, medications can have adverse side effects. By implementing holistic healing classes, therapists can give patients the foundation to reduce stress in their lives, thereby reducing the need for conventional medical treatment. Therapists can also experience both the benefits and the limitations of these approaches and find ways to use them with their patients. The clinical results may justify the investment of time and energy needed to embark on these extended courses of study [28].

The findings of our study have implications for advanced psycho-physiological practice, education and health care of patients with low sexual satisfaction and for others who suffer from sexual problems. The implementation of combined psycho-physiological therapy (stretching therapy combined with breathing exercise) classes taught by psycho-physiologists in outpatient clinics should be considered at the patient's first visit. A shift in emphasis from treating to teaching highlights the psychological function as a guide and teacher and makes patient care a more fulfilling partnership. All psychologists should have knowledge of holistic healing and implementation of combined psycho-physiological therapy (stretching therapy combined with breathing exercise), and classes in schools of psychology should introduce the concept of alternative healing therapies [29].

Our study has raised questions and has impacted other areas. Alternative and combined therapies suggest a wider vision of what medicine can and should be through appreciation of the interconnectedness of mind and body, emphasis on enhancing the body's own capacity for healing, and the use of the entire world's healing traditions. Alternative therapies can be used as adjuncts to treat all disease of the mind and the body. Thus, in less than a generation, the approaches and techniques currently called "alternative" may become an integral part of the practice of all family and sex therapy, psycho-physiology, and neuro-psychology practitioners.

Our study established combined psycho-physiological therapy (stretching therapy and breathing exercise) as being important to patients with family and sexual problems as it provides the patient with the power to decrease stress and to enhance the body's own capacity for healing. Integration of stretching therapy and breathing exercise in treatment plans allows a collaborative and democratic relationship between advanced psycho-physiological practitioners, other health providers, and the patients, who then reap the psychological and physiological rewards of feeling more in control of their lives.

### Limitations

These results are unique to this trial and will need to be replicated in future research. In this research, we had to use “convenience sampling” for the selection. The volunteers were selected from among men who visited the HUSM Family Clinic. Also, for data collection, we had to use the quasi-experimental design called the “non-equivalent control group.” In addition, the sample size might not be representative as patients were only from Kota Bharu (Kelantan, Malaysia), and the research was conducted within a limited time frame. Future researchers are encouraged to extend these results to other populations and to continue to pursue research on sexual dysfunction and marital satisfaction.

In this research, we obtained our subjects from among volunteers, after which the subjects were divided into two groups, the intervention and the control groups, regardless of their individual desires. However, after analyzing the groups’ baselines, we found them not to be the same. The volunteers in the intervention group seemed to have had more motivation to attend the intervention sessions and had, or felt that they had, experienced more difficulties than the volunteers in the control group For that reason, even though we found clear differences among the scores and the tests of the groups, those differences were not statistically significant. However, based on the results and the mean scores for the groups, the intervention clearly had a good effect on sexual satisfaction. Thus, we suggest future research with attention given to the motivation of the volunteers and to other methods of sampling. Also, additional research on the mechanisms by which psycho-physiological exercise and treatment improve family and sexual function, with emphasis on other populations and women, is needed.

### Endnotes

^1^HUSM: Hospital of Universiti Sains Malaysia.

^2^ENRICH: Enriching and Nutrition Relationship Issues, Communication and Happiness.

## Competing interests

The authors declare that they have no competing interests.

## Authors’ contributions

RB managed the research and carried out sampling, designed the therapy and the therapy sessions, performed the statistical analyses and design of the study, prepared instruments, carried out the pre-tests, the post-tests, the follow-up tests, and the data analyses, and wrote and approved the final manuscript. SBI participated in the official process by representing the authors before the ethical and research committee, participated in sample selection, participated in the management of the groups and the conduct of the study, and helped in the drafting, editing and coordination of the manuscript. WMZ and WNA Arifin participated in the statistical analyses. All authors read and approved the final manuscript.

## Pre-publication history

The pre-publication history for this paper can be accessed here:

http://www.biomedcentral.com/1471-2490/13/16/prepub
